# AI-Based Modeling: Techniques, Applications and Research Issues Towards Automation, Intelligent and Smart Systems

**DOI:** 10.1007/s42979-022-01043-x

**Published:** 2022-02-10

**Authors:** Iqbal H. Sarker

**Affiliations:** 1 Swinburne University of Technology, Melbourne, VIC 3122 Australia; 2 Department of Computer Science and Engineering, Chittagong University of Engineering & Technology, Chittagong, 4349 Bangladesh

**Keywords:** Artificial intelligence, Machine learning, Data science, Advanced analytics, Intelligent computing, Automation, Smart systems, Industry 4.0 applications

## Abstract

Artificial intelligence (AI) is a leading technology of the current age of the Fourth Industrial Revolution (Industry 4.0 or 4IR), with the capability of incorporating human behavior and intelligence into machines or systems. Thus, AI-based modeling is the key to build automated, intelligent, and smart systems according to today’s needs. To solve real-world issues, various types of AI such as analytical, functional, interactive, textual, and visual AI can be applied to enhance the intelligence and capabilities of an application. However, developing an effective AI model is a *challenging task* due to the dynamic nature and variation in real-world problems and data. In this paper, we present a comprehensive view on *“AI-based Modeling”* with the principles and capabilities of potential *AI techniques* that can play an important role in developing intelligent and smart systems in various *real-world application* areas including business, finance, healthcare, agriculture, smart cities, cybersecurity and many more. We also emphasize and highlight the *research issues* within the scope of our study. Overall, the goal of this paper is to provide a broad overview of AI-based modeling that can be used as a *reference guide* by academics and industry people as well as decision-makers in various real-world scenarios and application domains.

## Introduction

Nowadays, we live in a technological age, the Fourth Industrial Revolution, known as Industry 4.0 or 4IR [59, 91], which envisions fast change in technology, industries, societal patterns, and processes as a consequence of enhanced interconnectivity and smart automation. This revolution is impacting almost every industry in every country and causing a tremendous change in a non-linear manner at an unprecedented rate, with implications for all disciplines, industries, and economies. Three key terms *Automation*, i.e., reducing human interaction in operations, *Intelligent*, i.e., ability to extract insights or usable knowledge from data, and *Smart computing*, i.e., self-monitoring, analyzing, and reporting, known as self-awareness, have become fundamental criteria in designing today’s applications and systems in every sector of our lives since the current world is more reliant on technology than ever before. The use of modern smart technologies enables making smarter, faster decisions regarding the business process, ultimately increasing the productivity and profitability of the overall operation, where Artificial Intelligence (AI) is known as a leading technology in the area. The AI revolution, like earlier industrial revolutions that launched massive economic activity in manufacturing, commerce, transportation, and other areas, has the potential to lead the way of progress. As a result, the impact of AI on the fourth industrial revolution motivates us to focus briefly on “*AI-based modeling*” in this paper.

Artificial intelligence (AI) is a broad field of computer science concerned with building smart machines capable of performing tasks that typically require human intelligence. In other words, we can say that it aims is to make computers smart and intelligent by giving them the ability to think and learn using computer programs or machines, i.e., can think and function in the same way that people do. From a philosophical perspective, AI has the potential to help people live more meaningful lives without having to work as hard, as well as manage the massive network of interconnected individuals, businesses, states, and nations in a way that benefits everyone. Thus, the primary goal of AI is to enable computers and machines to perform cognitive functions such as problem-solving, decision making, perception, and comprehension of human communication. Therefore, AI-based modeling is the key to building automated, intelligent and smart systems according to today’s needs, which has emerged as the next major technological milestone, influencing the future of practically every business by making every process better, faster, and more precise.

While today’s Fourth Industrial Revolution is typically focusing on technology-driven “automation, intelligent and smart systems”, AI technology has become one of the core technologies to achieve the goal. However, developing an effective AI model is a challenging task due to the dynamic nature and variation in real-world problems and data. Thus, we take into account several AI categories: The first one is “*Analytical AI*” with the capability of extracting insights from data to ultimately produce recommendations and thus contributing to data-driven decision-making; the Second one is “*Functional AI*” which is similar to analytical AI; however, instead of giving recommendations, it takes actions; the Third one is “*Interactive AI*” that typically allows businesses to automate communication without compromising on interactivity like smart personal assistants or chatbots; the Fourth one is “*Textual AI*” that covers textual analytics or natural language processing through which business can enjoy text recognition, speech-to-text conversion, machine translation, and content generation capabilities; and finally the Fifth one is “*Visual AI*” that covers computer vision or augmented reality fields, discussed briefly in “Why artificial intelligence in today’s research and applications?”.

Although the area of “artificial intelligence” is huge, we mainly focus on potential techniques towards solving real-world issues, where the results are used to build automated, intelligent, and smart systems in various application areas. To build AI-based models, we classify various AI techniques into ten categories: (1) machine learning; (2) neural networks and deep learning; (3) data mining, knowledge discovery and advanced analytics; (4) rule-based modeling and decision-making; (5) fuzzy logic-based approach; (6) knowledge representation, uncertainty reasoning, and expert system modeling; (7) case-based reasoning; (8) text mining and natural language processing; (9) visual analytics, computer vision and pattern recognition; (10) hybridization, searching and Optimization. These techniques can play an important role in developing intelligent and smart systems in various *real-world application* areas that include business, finance, healthcare, agriculture, smart cities, cybersecurity, and many more, depending on the nature of the problem and target solution. Thus, it is important to comprehend the concepts of these techniques mentioned above, as well as their relevance in a variety of real-world scenarios, discussed briefly in “Potential AI techniques”.

Based on the importance and capabilities of AI techniques, in this paper, we give a comprehensive view on “AI-based modeling” that can play a key role towards automation, intelligent and smart systems according to today’s needs. Thus, the key focus is to explain the principles of various AI techniques and their applicability to the advancement of computing and decision-making to meet the requirements of the Fourth Industrial Revolution. Therefore the purpose of this paper is to provide a fundamental guide for those academics and industry professionals who want to study, research, and develop automated, intelligent, and smart systems based on artificial intelligence techniques in relevant application domains.

The main contributions of this paper are therefore listed as follows:

- To define the scope of our study in terms of automation, intelligent and smart computing, and decision-making in the context of today’s real-world needs.
- To explore various types of AI that includes analytical, functional, interactive, textual, and visual AI, to understand the theme of the power of artificial intelligence in computing and decision-making while solving various problems in today’s Fourth Industrial Revolution.
- To provide a comprehensive view on AI techniques that can be applied to build an AI-based model to enhance the intelligence and capabilities of a real-world application.
- To discuss the applicability of AI-based solutions in various real-world application domains to assist developers as well as researchers in broadening their perspectives on AI techniques.
- To highlight and summarize the potential research issues within the scope of our study for conducting future research, system development and improvement.

The rest of the paper is organized as follows. The next section provides a background highlighting why artificial intelligence is in today’s research and application. In the subsequent section, we discuss and summarize how various AI techniques can be used for intelligence modeling in various application areas. Next, we summarize various real-world application areas, where AI techniques can be employed to build automated, intelligent, and smart systems. The impact and future aspect of AI highlighting research issues have been presented in the penultimate section, and the final section concludes this paper.

## Why Artificial Intelligence in Today’s Research and Applications?

In this section, our goal is to motivate the study of various AI techniques that can be applied in various application areas in today’s interconnected world. For this, we explore Industry 4.0 and the revolution of AI, types of AI techniques, as well as the relation with the most prominent machine and deep learning techniques. Hence, the scope of our study in terms of research and applications is also explored through our discussion.

### Industry 4.0 and the Revolution of AI

We are now in the age of the 4th Industrial Revolution, referred to as Industry 4.0 [59, 91], which represents a new era of innovation in technology, particularly, AI-driven technology. After the Internet and mobile Internet sparked the 3rd Industrial Revolution, AI technologies, fueled by data, are now creating an atmosphere of Industry 4.0. The term “Industry 4.0” typically refers to the present trend of leveraging modern technology to automate processes and exchange information. In a broad sense, Industry 4.0 has been defined as “A term used to describe the present trend of industrial technology automation and data exchange, which includes cyber-physical systems, the Internet of Things, cloud computing, and cognitive computing, as well as the development of the smart factory”. The digital revolution to Industry 4.0 begins with data collection, followed by artificial intelligence to interpret the data. Thus, the term “Intelligence Revolution” can be considered in the context of computing and services as the world is being reshaped by AI that incorporates human behavior and intelligence into machines or systems.

AI is the buzzword these days as it is going to impact businesses of all shapes and sizes, across all industries. Existing products or services can be enhanced by industrial AI to make them more effective, reliable, and safe. For example, computer vision is used in the automotive industry to avoid collisions and allow vehicles to stay in their lane, making driving safer. The world’s most powerful nations are hurrying to jump on the AI bandwagon and are increasing their investments in the field. Similarly, the largest and most powerful corporations are working hard to build ground-breaking AI solutions that will put them ahead of the competition. As a result, its impact may be observed in practically every area including homes, businesses, hospitals, cities, and the virtual world, as summarized in “Real-World Applications of AI”.

**Fig. 1:**
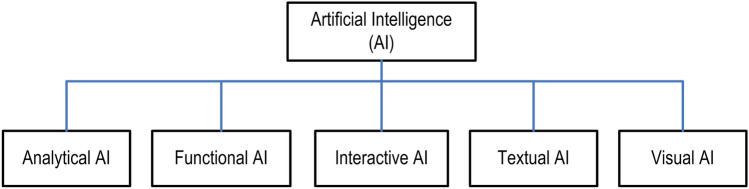
Various types of artificial intelligence (AI) considering the variations of real-world issues

### Understanding Various Types of Artificial Intelligence

Artificial intelligence (AI) is primarily concerned with comprehending and carrying out intelligent tasks such as thinking, acquiring new abilities, and adapting to new contexts and challenges. AI is thus considered a branch of science and engineering that focuses on simulating a wide range of issues and functions in the field of human intellect. However, due to the dynamic nature and diversity of real-world situations and data, building an effective AI model is a challenging task. Thus, to solve various issues in today’s Fourth Industrial Revolution, we explore various types of AI that include analytical, functional, interactive, textual, and visual, to understand the theme of the power of AI, as shown in Fig. 1. In the following, we define the scope of each category in terms of computing and real-world services.

- *Analytical AI:* Analytics typically refers to the process of identifying, interpreting, and communicating meaningful patterns of data. Thus, Analytical AI aims to discover new insights, patterns, and relationships or dependencies in data and to assist in data-driven decision-making. Therefore, in the domain of today’s business intelligence, it becomes a core part of AI that can provide insights to an enterprise and generate suggestions or recommendations through its analytical processing capability. Various machine learning [81] and deep learning [80] techniques can be used to build an analytical AI model to solve a particular real-world problem. For instance, to assess business risk, a data-driven analytical model can be used.
- *Functional AI:* Functional AI works similarly to analytical AI because it also explores massive quantities of data for patterns and dependencies. Functional AI, on the other hand, executes actions rather than making recommendations. For instance, a functional AI model could be useful in robotics and IoT applications to take immediate actions.
- *Interactive AI:* Interactive AI typically enables efficient and interactive communication automation, which is well established in many aspects of our daily lives, particularly in the commercial sphere. For instance, to build chatbots and smart personal assistants an interactive AI model could be useful. While building an interactive AI model, a variety of techniques such as machine learning, frequent pattern mining, reasoning, AI heuristic search can be employed.
- *Textual AI:* Textual AI typically covers textual analytics or natural language processing through which businesses can enjoy text recognition, speech-to-text conversion, machine translation as well as content generation capabilities. For instance, an enterprise may use textual AI to support an internal corporate knowledge repository to provide relevant services, e.g., answering consumers’ queries.
- *Visual AI:* Visual AI is typically capable to recognize, classify, and sorting items, as well as converting images and videos into insights. Thus, visual AI can be considered as a branch of computer science that trains machines to learn images and visual data in the same manner that humans do. This sort of AI is often used in fields such as computer vision and augmented reality.

As discussed above, each of the AI types has the potential to provide solutions to various real-world problems. However, to provide solutions by taking into account the target applications, various AI techniques and their combinations that include machine learning, deep learning, advanced analytics, knowledge discovery, reasoning, searching, and relevant others can be used, discussed briefly in “Potential AI techniques”. As most of the real-world issues need advanced analytics [79] to provide an intelligent and smart solution according to today’s needs, analytical AI that uses machine learning (ML) and deep learning (DL) techniques can play a key role in the area of AI-powered computing and system.

### The Relation of AI with ML and DL

**Fig. 2:**
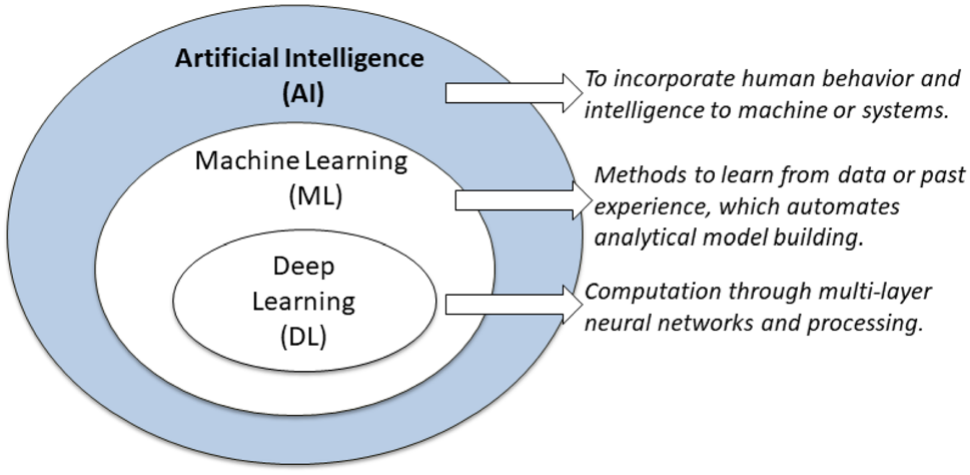
An illustration of the position of machine learning (ML) and deep Learning (DL) within the area of artificial intelligence (AI)

Artificial intelligence (AI), machine learning (ML), and deep learning (DL) are three prominent terminologies used interchangeably nowadays to represent intelligent systems or software. The position of machine learning and deep learning within the artificial intelligence field is depicted in Fig. 2. According to Fig. 2, DL is a subset of ML which is also a subset of AI. In general, AI [77] combines human behavior and intelligence into machines or systems, whereas ML is a way of learning from data or experience [81], which automates analytical model building. Deep learning [80] also refers to data-driven learning approaches that use multi-layer neural networks and processing to compute. In the deep learning approach, the term “Deep” refers to the concept of numerous levels or stages through which data is processed to develop a data-driven model.

Thus, both ML and DL can be considered as essential AI technologies, as well as a frontier for AI that can be used to develop intelligent systems and automate processes. It also takes AI to a new level, termed “Smarter AI” with data-driven learning. There is a significant relationship with “Data Science” [79] as well because both ML and DL can learn from data. These learning methods can also play a crucial role in advanced analytics and intelligent decision-making in data science, which typically refers to the complete process of extracting insights in data in a certain problem domain. Overall, we can conclude that both ML and DL technologies have the potential to transform the current world, particularly in terms of a powerful computational engine, and to contribute to technology-driven automation, smart and intelligent systems. In addition to these learning techniques, several others can play the role in the development of AI-based models in various real-world application areas, depending on the nature of the problem and the target solution, discussed briefly in “ Potential AI techniques”.

## Potential AI Techniques

In this section, we briefly discuss the principles and capabilities of potential AI techniques that can be used in developing intelligent and smart systems in various real-world application areas. For this we divide AI techniques into ten potential categories by taking into account various types of AI, mentioned in earlier “Why artificial intelligence in today’s research and applications?”. Followings are the ten categories of AI techniques that can play a key role in automation, intelligent, and smart computer systems, depending on the nature of the problem.

### Machine Learning

**Fig. 3:**
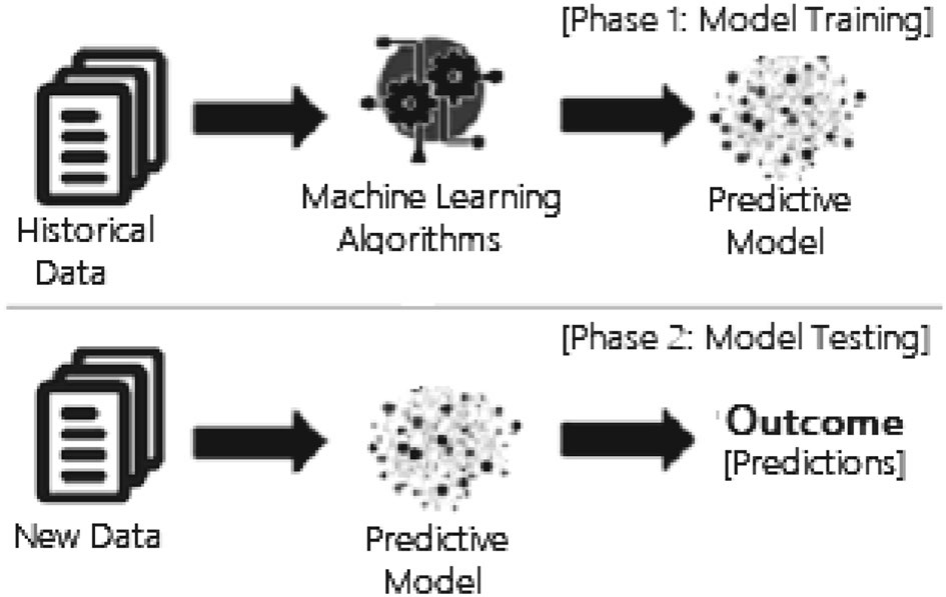
A general structure of a machine learning based predictive model considering both the training and testing phase

Machine learning (ML) is known as one of the most promising AI technologies, which is typically the study of computer algorithms that automate analytical model building [81]. ML models are often made up of a set of rules, procedures, or sophisticated “transfer functions” that can be used to discover interesting data patterns or anticipate behavior [23]. Machine learning is also known as predictive analytics that makes predictions about certain unknowns in the future through the use of data and is used to solve many real-world business issues, e.g., business risk prediction. In Fig. 3, a general framework of a machine learning-based predictive model is depicted, where the model is trained from historical data in phase 1 and the outcome is generated for new test data in phase 2. For modeling in a particular problem domain, different types of machine learning techniques can be used according to their learning principles and capabilities, as discussed below.

**Table 1:** Various types of machine learning techniques with examples

Learning type	Model building	Tasks
Supervised	Algorithms or models learn from labeled data (Task-Driven Approach)	Classification, Regression
Unsupervised	Algorithms or models learn from unlabeled data (Data-Driven Approach)	Clustering, Associations, Dimensionality Reduction
Semi-supervised	Models are built using combined data (Labeled + Unlabeled)	Classification, Clustering
Reinforcement	Models are based on reward or penalty (Environment-Driven Approach)	Classification, Control

- *Supervised learning* This is performed when particular goals are specified to be achieved from a set of inputs, i.e., a ‘task-driven strategy’ that uses labeled data to train algorithms to classify data or forecast outcomes, for example—detecting spam-like emails. The two most common supervised learning tasks are classification (predicting a label) and regression (predicting a quantity) analysis, discussed briefly in our earlier paper Sarker et al. [81]. Navies Bayes [42], K-nearest neighbors [4], Support vector machines [46], Decision Trees - ID3 [71], C4.5 [72], CART [15], BehavDT [84], IntrudTree [82], Ensemble learning, Random Forest [14], Linear regression [36], Support vector regression [46], etc. [81] are the popular techniques that can be used to solve various supervised learning tasks, according to the nature of the given data in a particular problem domain. For instance, to detect various types of cyber-attacks the classification models could be useful, while cyber-crime trend analysis or estimating the financial loss in the domain of cybersecurity, a regression model could be useful, which enables enterprises to assess and manage their cyber-risk.
- *Unsupervised learning* This is referred to as a ‘data-driven method’, in which the primary goal is to uncover patterns, structures, or knowledge from unlabeled data. Clustering, visualization, dimensionality reduction, finding association rules, and anomaly detection are some of the most common unsupervised tasks, discussed briefly in our earlier paper Sarker et al. [81]. The popular techniques for solving unsupervised learning tasks are clustering algorithms such as K-means [55], K-Mediods [64], CLARA [45], DBSCAN [27], hierarchical clustering, single linkage [92] or complete linkage [93], BOTS [86], association learning algorithms such as AIS [2], Apriori [3], Apriori-TID and Apriori-Hybrid [3], FP-Tree [37], and RARM [18], Eclat [105], ABC-RuleMiner [88] as well as feature selection and extracting techniques like Pearson Correlation [81], principal component analysis [40, 66], etc. that can be used to solve various unsupervised learning-related tasks, according to the nature of the data. An unsupervised clustering model, for example, could be useful in customer segmentation or identifying different consumer groups around which to build marketing or other business strategies.
- *Other learning techniques* In addition to particular supervised and unsupervised tasks, semi-supervised learning can be regarded as a hybridization of both techniques explained above, as it uses both labeled and unlabeled data to train a model. It could be effective for improving model performance when data must be labeled automatically without human interaction. For instance, classifying Internet content or texts, a semi-supervised learning model could be useful. Reinforcement learning is another type of machine learning training strategy that rewards desired behaviors while punishing unwanted ones. A reinforcement learning agent, in general, is capable of perceiving and interpreting its surroundings, taking actions, and learning through trial and error, i.e., an environment-driven approach, in which the environment is typically modeled as a Markov decision process and decisions are made using a reward function [10]. Monte Carlo learning, Q-learning, Deep Q Networks, are the most common reinforcement learning algorithms [43]. Trajectory optimization, motion planning, dynamic pathing, and scenario-based learning policies for highways are some of the autonomous driving activities where reinforcement learning could be used.

Overall, machine learning modeling [81] has been employed in practically every aspect of our lives, including healthcare, cybersecurity, business, education, virtual assistance, recommendation systems, smart cities, and many more. Blumenstock et al. [12], for example, provides a machine learning strategy for getting COVID-19 assistance to people who need it the most. Sarker et al. highlight numerous sorts of cyber anomalies and attacks that can be detected using machine learning approaches in the domain of cybersecurity [78, 89]. Saharan et al. [76] describe a machine-learning-based strategy to develop an effective smart parking pricing system for smart city environments. In our earlier paper, Sarker et al. [81] we briefly discussed various types of machine learning techniques including clustering, feature learning, classification, regression, association analysis, etc. highlighting their working principles, learning capabilities, and real-world applications. In Table 1, we have outlined the above-mentioned machine learning techniques, emphasizing model building procedures and tasks. Overall, machine learning algorithms can build a model based on training data of a particular problem domain, to make predictions or decisions without having to be explicitly programmed to do so. Thus, we can conclude that machine learning approaches can play a crucial part in the development of useful models in a variety of application areas, based on their learning capabilities and the nature of the data, and the desired outcome.

### Neural Networks and Deep Learning

**Fig. 4:**
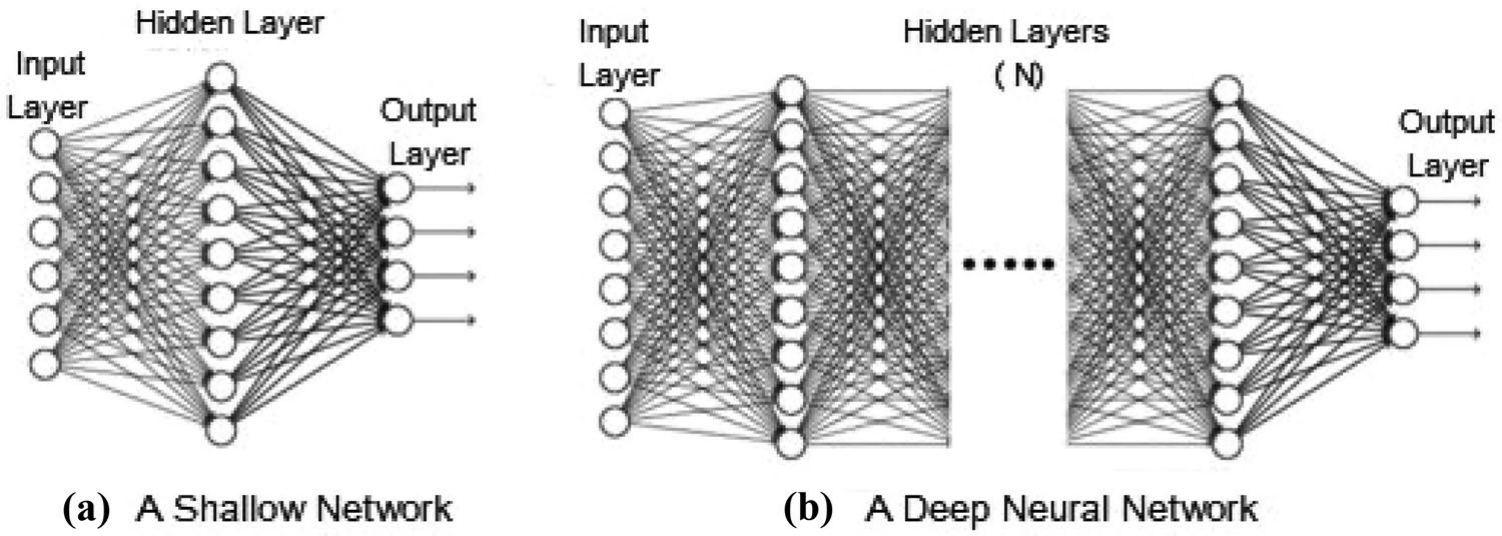
A general architecture of **a** a shallow network with one hidden layer and **b** a deep neural network with multiple hidden layers

Deep learning (DL) [80] is known as another popular AI technique, which is based on artificial neural networks (ANN). Nowadays, DL has become a hot topic in the computing world due to its layer-wise learning capability from data. Multiple hidden layers, including input and output layers, make up a typical deep neural network. Figure 4 shows a general structure of a deep neural network ($hidden \; layer=N$ and N $\ge$ 2) comparing with a shallow network ($hidden \; layer=1$). DL techniques can be divided into three major categories, highlighted in our earlier paper Sarker et al. [80]. These are as below:

**Fig. 5:**
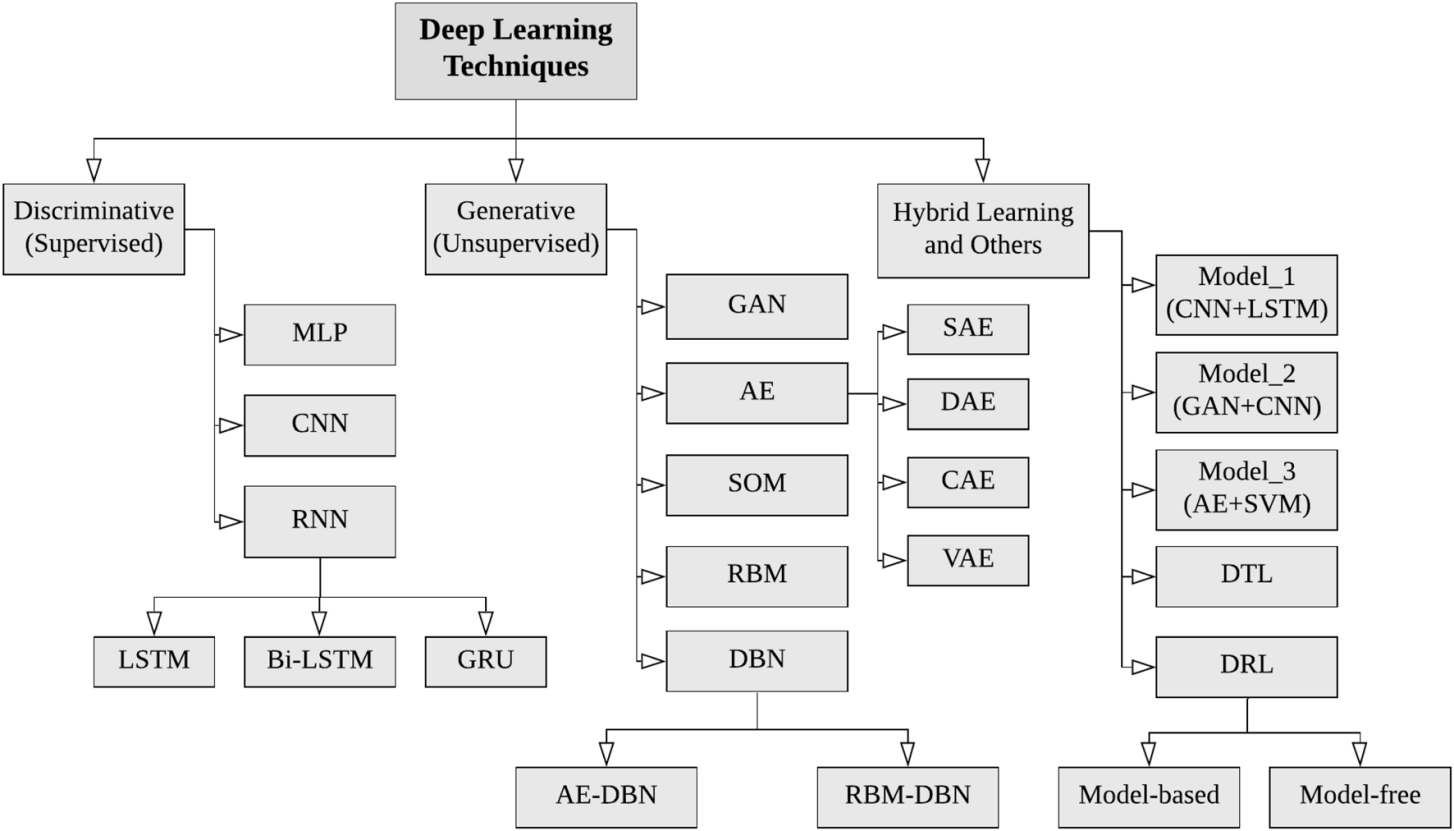
A taxonomy of DL techniques [80], broadly divided into three major categories (1) deep networks for supervised or discriminative learning, (2) deep networks for unsupervised or generative learning, and (3) deep networks for hybrid learning and relevant others

- *Deep networks for supervised or discriminative learning* In supervised or classification applications, this type of DL approach is used to provide a discriminative function. Discriminative deep architectures are often designed to provide pattern categorization discrimination by characterizing the posterior distributions of classes conditioned on observable data [20]. Multi-layer perceptron (MLP) [67], Convolutional neural networks (CNN or ConvNet) [53], Recurrent neural networks (RNN) [24, 57], and their variants can be used to build the deep discriminative learning models to solve the relevant real-world issues.
- *Deep networks for unsupervised or generative learning* This category of deep learning approaches is commonly used to identify high-order correlation qualities or features for pattern analysis or synthesis, as well as the joint statistical distributions of visible data and their associated classes [20]. The key notion of generative deep architectures is that specific supervisory information, such as target class labels, is unimportant throughout the learning process. Techniques in this category are mostly employed for unsupervised learning, as they are commonly used for feature learning or data generation and representation [19, 20]. Thus, generative modeling can also be utilized as a preprocessing step for supervised learning tasks, ensuring discriminative model accuracy. The Generative Adversarial Network (GAN) [32], Autoencoder (AE) [31], Restricted Boltzmann Machine (RBM) [58], Self-Organizing Map (SOM) [50], and Deep Belief Network (DBN) [39], as well as their variants, can be used to build the deep generative learning models to solve the relevant real-world issues.
- *Deep networks for hybrid learning* Generative models are versatile, learning from both labeled and unlabeled data. In contrast, discriminative models are unable to learn from unlabeled data yet outperform their generative versions in supervised tasks. Hybrid networks are motivated by a paradigm for simultaneously training deep generative and discriminative models. Multiple (two or more) deep basic learning models make up hybrid deep learning models, with the basic model being the discriminative or generative deep learning model outlined previously. For instance, a generative model followed by a discriminative model, or an integration of a generative or discriminative model followed by a non-deep learning classifier, may be effective for tackling real-world problems.

Figure 5 shows a taxonomy of these DL techniques that can be employed in many application areas including healthcare, cybersecurity, business, virtual help, smart cities, visual analytics, and many more. For example, Aslan et al. [9] offer a CNN-based transfer learning strategy for COVID-19 infection detection. Islam et al. [41] describes a combined deep CNN-LSTM network for the identification of novel coronavirus (COVID-19) using X-ray images. Using transferable generative adversarial networks built on deep autoencoders, Kim et al. [48] propose a method for detecting zero-day malware. Anuradha et al. [8] propose a deep CNN-based stock trend prediction utilizing a reinforcement-LSTM model based on large data. Wang et al. [100] offer a real-time collision prediction technique for intelligent transportation systems based on deep learning. Dhyani et al. [22] proposed an intelligent Chatbot utilizing deep learning with Bidirectional RNN and attention model. Overall, deep learning approaches can play a crucial role in the development of effective AI models in a variety of application areas, based on their learning capabilities and the nature of the data, and the target outcome.

### Data Mining, Knowledge Discovery and Advanced Analytics

**Fig. 6:**
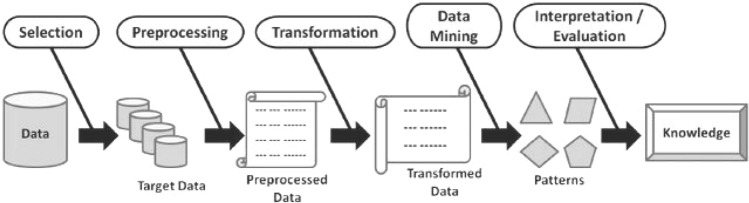
A general procedure of the knowledge discovery process

Over the last decade, data mining has been a common word that is interchangeable with terms like knowledge mining from data, knowledge extraction, knowledge discovery from data (KDD), data or pattern analysis, etc. [79]. Figure 6 shows a general procedure of the knowledge discovery process. According to Han et al. [36], the term “knowledge mining from data” should have been used instead. Data mining is described as the process of extracting useful patterns and knowledge from huge volumes of data [36], which is related to another popular term “Data Science” [79]. Data science is typically defined as a concept that unites statistics, data analysis, and related methodologies to analyze and investigate realities through data.

**Table 2:** Various types of analytical methods with examples

Analytical methods	Data-driven model building	Examples
Descriptive Analytics	Answer the question, “what happened in the past”?	Summarising past events, e.g., sales, business data, social media usage, reporting general trends, etc.
Diagnostic Analytics	Answer the question, “why did it happen?”	Identify anomalies and determine casual relationships, to find out business loss, identifying the influence of medications, etc.
Predictive Analytics	Answer the question, “what will happen in the future?”	Predicting customer preferences, recommending products, identifying possible security breaches, predicting staff and resource needs, etc.
Prescriptive Analytics	Answer the question, “what action should be taken?”	Improving business management, maintenance, improving patient care and healthcare administration, determining optimal marketing strategies, etc.

In the area of data analytics, several key questions such as “What happened?”, “Why did it happen?”, “What will happen in the future?”, “What action should be taken?” are common and important [79]. Based on these questions, four types of analytics such as descriptive, diagnostic, predictive, and prescriptive analytics are highlighted below, which can be used to build the corresponding data-driven models.

- *Descriptive analytics* It is the analysis of historical data to have a better understanding of how a business has changed. Thus, descriptive analytics answers the question, “What happened in the past?” by describing historical data such as sales and operations statistics, marketing tactics, social media usage, etc.
- *Diagnostic analytics* It is a type of sophisticated analytics that explores data or content to figure out “Why did it happen?” The purpose of diagnostic analytics is to assist in the discovery of the problem’s root cause.
- *Predictive analytics* This type of advanced analytics typically explores data to answer the question, “What will happen in the future?” Thus, the primary purpose of predictive analytics is to identify and, in most cases, answer this question with a high degree of confidence.
- *Prescriptive analytics* This focuses on advising the optimal course of action based on data to maximize the total outcomes and profitability, answering the question, “What action should be taken?”

To summarize, both descriptive and diagnostic analytics examine the past to determine what happened and why it happened. Predictive and prescriptive analytics employ historical data to foresee what will happen in the future and what actions should be made to mitigate such impacts. For a clear understanding, Table 2 shows a summary of these analytics that are applied in various application areas. For example, Hamed et al. [35] build decision support systems in Arabic higher education institutions using data mining and business intelligence. Alazab et al. [5] provide a data mining strategy to maximize the competitive advantage on E-business websites. From logs to stories, Afzaliseresht et al. [1] provide human-centered data mining for cyber threat information. Poort et al. [70] have described an automated diagnostic analytics workflow for the detection of production events-application to mature gas fields. Srinivas et al. [94] provide a prescriptive analytics framework for optimizing outpatient appointment systems using machine learning algorithms and scheduling rules. Thus, we can conclude data mining and analytics can play a crucial part to build AI models through the extracted insights from the data.

### Rule-Based Modeling and Decision-Making

Typically, a rule-based system is used to store and modify knowledge to understand data in a meaningful way. A rule base is a sort of knowledge base that has a list of rules. In most cases, rules are written as IF-THEN statements of the form:

IF $$<antecedent>$$ THEN $$<consequent>$$

Such an IF-THEN rule-based expert system model can have the decision-making ability of a human expert in an intelligent system designed to solve complex problems and knowledge reasoning [85]. The reason is that the rules in professional frameworks are easily understood by humans and are capable of representing relevant knowledge clearly and effectively. Furthermore, rule-based models may be quickly improved according to the demands by adding, deleting, or updating rules based on domain expert information, or recency, i.e. based on recent trends [83].

Previously, the term “rule-based system” was used to describe systems that used rule sets that were handcrafted or created by humans. However, rule-based machine learning approaches could be more effective in terms of automation and intelligence, which include mainly classification and association rule learning techniques [85]. Several popular classification techniques such as decision trees [72], IntrudTree [82], BehavDT [84], Ripple Down Rule learner (RIDOR) [101], Repeated Incremental Pruning to Produce Error Reduction (RIPPER) [102], etc. exist with the ability of rule generation. Based on support and confidence value, association rules are built by searching for frequent IF-THEN pattern data. Common association rule learning techniques such as AIS [2], Apriori [3], FP-Tree [37], RARM [18], Eclat [105], ABC-RuleMiner [88], and others can be used to build a rule-based model utilizing a given data set. Sarker et al. [88], for example, provide a rule-based machine learning strategy for context-aware intelligent and adaptive mobile services. Borah et al. [13] propose a method for employing dynamic rare association rule mining to find risk variables for unfavorable illnesses. Using case-based clustering and weighted association rule mining, Bhavithra et al. [11] offer a personalized web page suggestion. Xu et al. [103] introduced a risk prediction and early warning system for air traffic controllers’ risky behaviors utilizing association rule mining and random forest. Thus, we can conclude that rule-based modeling can play a significant role to build AI models as well as intelligent decision-making in various application areas to solve real-world issues.

### Fuzzy Logic-Based Approach

**Fig. 7:**
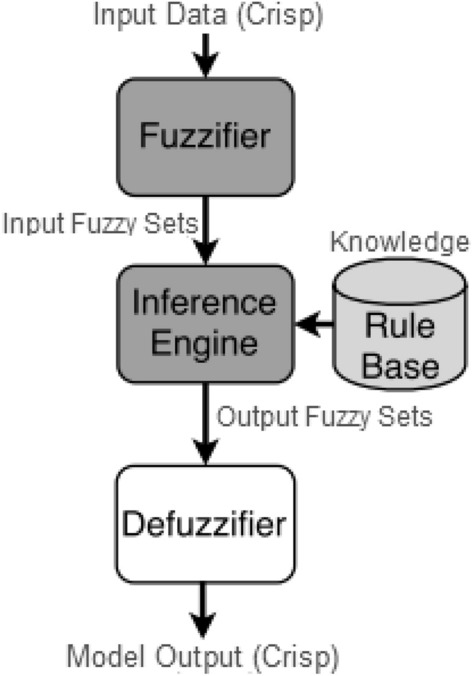
A general architecture of fuzzy logic systems

Fuzzy logic is a precise logic of imprecision and approximate reasoning [104]. This is a natural generalization of standard logic in which a concept’s degree of truth, also known as membership value or degree of membership, can range from 0.0 to 1.0. Standard logic only applies to concepts that are either completely true, i.e., degree of truth 1.0, or completely false, i.e., degree of truth 0.0. Fuzzy logic, on the other hand, has been used to deal with the concept of partial truth, in which the truth value may range from completely true to completely false, such as 0.9 or 0.5. For instance, “if x is very large, do y; if x is not very large, do z”. Here the boundaries of very big and not too big may overlap, i.e. fuzzy. As a result, fuzzy logic-based models can recognize, represent, manipulate, understand, and use data and information that are vague and uncertain [104]. Figure 7 shows a general architecture of a fuzzy logic system. It typically has four parts as below:

- *Fuzzification* It transforms inputs, i.e. crisp numbers into fuzzy sets.
- *Knowledge-base* It contains the set of rules and the IF-THEN conditions provided by the experts to govern the decision-making system, based on linguistic information.
- *Inference engine* It determines the matching degree of the current fuzzy input concerning each rule and decides which rules are to be fired according to the input field. Next, the fired rules are combined to form the control actions.
- *Defuzzification* It transforms the fuzzy sets obtained by the inference engine into a crisp value.

Although machine learning models are capable of differentiating between two (or more) object classes based on their ability to learn from data, the fuzzy logic approach is preferred when distinguishing features are vaguely defined and rely on human expertise and knowledge. Thus, the system may work with any type of input data, including imprecise, distorted, or noisy data, as well as with limited data. It is a suitable strategy to use in scenarios with real, continuous-valued elements because it uses data acquired in surroundings with such properties [34]. Fuzzy logic-based models are used to tackle problems in a variety of fields. Reddy et al. [74], for example, use a fuzzy logic classifier for heart disease detection, with the derived rules from fuzzy classifiers being optimized using an adaptive genetic algorithm. Krishnan et al. [51] describes a fuzzy logic-based smart irrigation system using IoT, which sends out periodic acknowledgment messages on task statuses such as soil humidity and temperature. Hamamoto et al. [34] describe a network anomaly detection method based on fuzzy logic for determining whether or not a given instance is anomalous. Kang et al. [44] proposed a fuzzy weighted association rule mining approach for developing a customer satisfaction product form. Overall, we can infer that fuzzy logic can make reasonable conclusions in a world of imprecision, uncertainty, and partial data, and thus might be useful in such scenarios while building a model.

### Knowledge Representation, Uncertainty Reasoning, and Expert System Modeling

Knowledge representation is the study of how an intelligent agent’s beliefs, intents, and judgments may be expressed appropriately for automated reasoning, and it has emerged as one of the most promising topics of Artificial Intelligence. Reasoning is the process of using existing knowledge to conclude, make predictions, or construct explanations. Many types of knowledge can be used in various application domains include descriptive knowledge, structural knowledge, procedural knowledge, meta-knowledge, and heuristic knowledge [87]. Knowledge representation is more than just storing data in a database; it also allows an intelligent machine to learn from its knowledge and experiences to act intelligently as a human. As a result, in designing an intelligent system, an effective method of knowledge representation is required. Several knowledge representation approaches exist in the fields that can be utilized to develop a knowledge-based conceptual model, including logical, semantic network, frame, and production rules [95]. In the following, we summarize the potential knowledge representation strategies taking real-world issues into account.

**Fig. 8:**
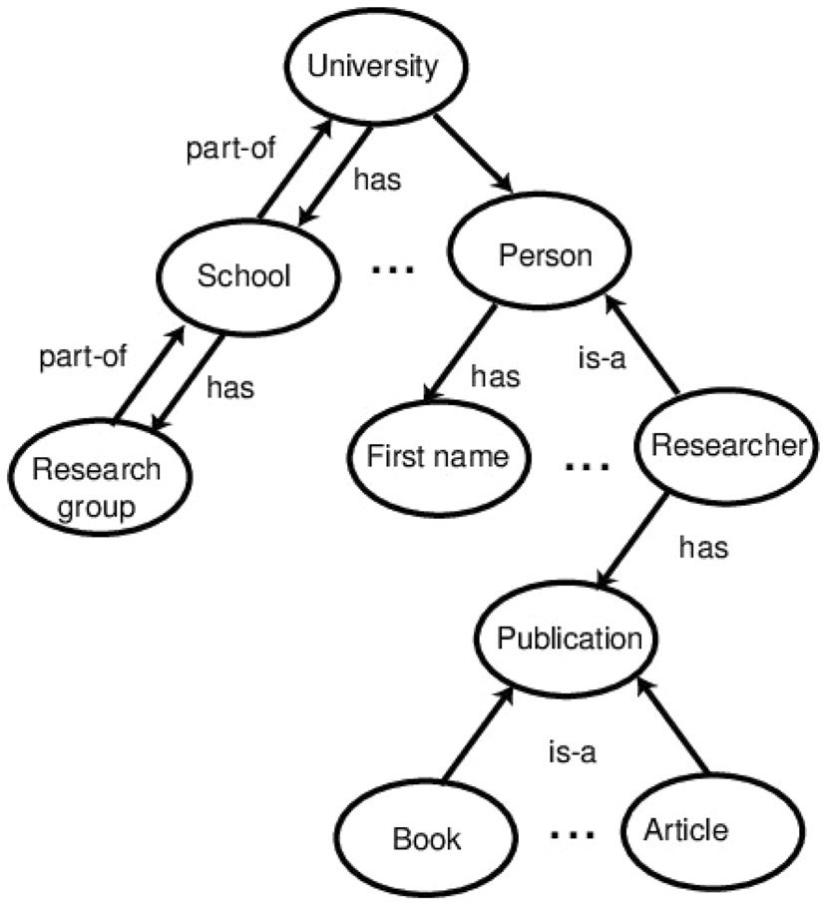
An example of ontology components for the entity University [26]

**Fig. 9:**
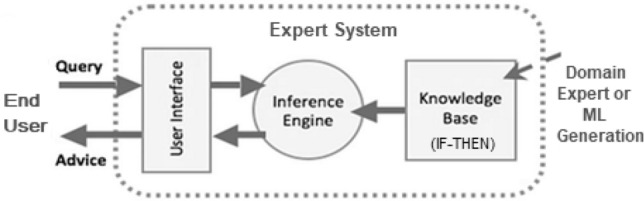
A general architecture of an expert system

- *Ontology-based* In general, ontology is “an explicit specification of conceptualization and a formal way to define the semantics of knowledge and data” [56]. According to [56], formally, an ontology is represented as “$\{O = C, R, I, H, A\}$, where $\{C = C_1, C_2,\ldots ,C_n\}$ represents a set of concepts, and $\{R = R_1, R_2,\ldots ,R_m\}$ represents a set of relations defined over the concepts. *I* represents a set of instances of concepts, and *H* represents a Directed Acyclic Graph (DAG) defined by the subsumption relation between concepts, and *A* represents a set of axioms bringing additional constraints on the ontology”. Ontology-based knowledge representation and reasoning techniques provide sophisticated knowledge about the environment for processing tasks or methods. Figure 8 shows an example of ontology components for the entity University [26]. By defining shared and common domain theories, ontologies help people and machines to communicate concisely by supporting semantic knowledge for a particular domain. In the area of semantic data mining, such ontology-based approaches like classification, mining with association rules, clustering, finding links, etc. can play a significant role to build smart systems.
- *Rule-base* It typically consists of pairs of the condition, and corresponding action, which means, “IF $<condition>$ THEN $<action>$” [85]. As a result, an agent checks the condition first, and if the condition is satisfied, the related rule fires. The key benefit of a rule-based system like this is that the “condition” part can select which rule is appropriate to use for a given scenario. The “action” portion, on the other hand, is responsible for implementing the problem’s solutions. Furthermore, in a rule-based system, we can easily insert, delete, or update rules as needed.
- *Uncertainty and probabilistic reasoning* Probabilistic reasoning is a method of knowledge representation in which the concept of probability is used to signify the uncertainty in knowledge, and where probability theory and logic are combined to address the uncertainty [65]. Probability is the numerical measure of the possibility of an event occurring, and it can be defined as the chance that an uncertain event will occur. To deal with uncertainty in a model, probabilistic models, fuzzy logic, Bayesian belief networks, etc. can be employed.

A knowledge-based system, such as an expert system for decision-making, relies on these representations of knowledge. The inference engine and the knowledge base are two subsystems of the expert system, as represented in Fig. 9. The information in the knowledge base is organized according to the knowledge representation discussed above. The inference engine looks for knowledge-based information and linkages and, like a human expert, provides answers, predictions, and recommendations. Such a knowledge-based system can be found in many application areas. For instance, Goel et al. [29] present an ontology-driven context-aware framework for smart traffic monitoring. Chukkapalli et al. [16] present ontology-driven AI and access control systems for smart fisheries. Kiran et al. [49] present enhanced security-aware technique and ontology data access control in cloud computing. Syed et al. [97] present a conceptual ontology and cyber intelligence alert system for cybersecurity vulnerability management. An ontology-based cyber security policy implementation in Saudi Arabia has been presented in Talib et al. [98]. Recently, Sarker et al. [90] explores an expert system modeling for personalized decision-making in mobile apps. Thus, knowledge representation and modeling are important to build AI models as well as intelligent decision-making in various application areas to solve real-world issues.

### Case-Based Reasoning

**Fig. 10:**
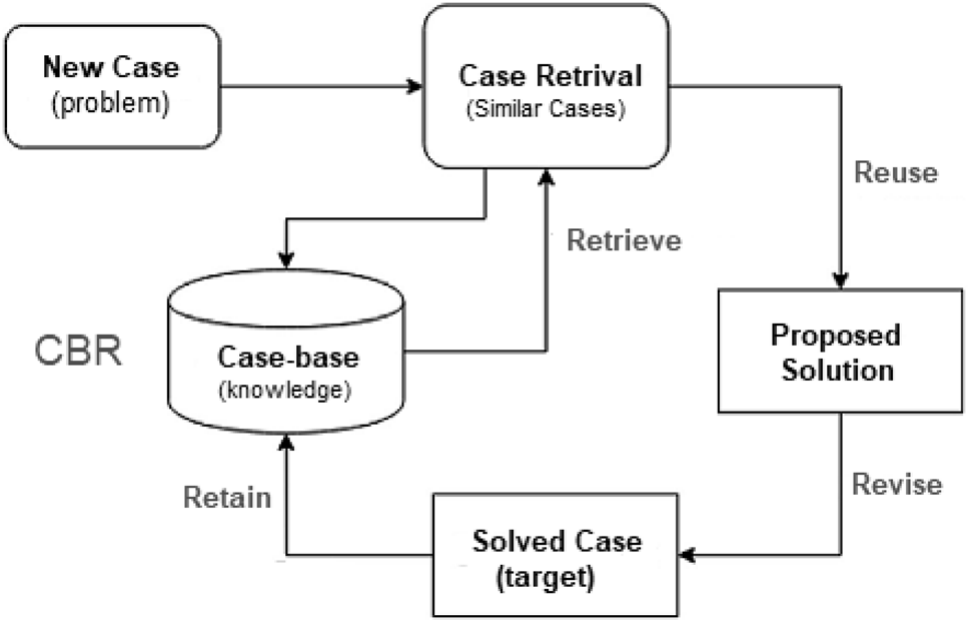
A general architecture of case-based reasoning

Case-based reasoning (CBR) is a cognitive science and AI paradigm that represents reasoning as primarily memory-based. CBR is concerned with the “smart” reuse of knowledge from previously solved problems (“cases”) and its adaption to new and unsolved problems. The inference is a problem-solving strategy based on the similarity of the current situation to previously solved problems recorded in a repository. Its premise is that the more similar the two issues are, the more similar their solutions will be. Thus, case-based reasoners handle new problems by obtaining previously stored ’cases’ that describe similar earlier problem-solving experiences and customizing their solutions to meet new requirements. For example, patient case histories and treatments are utilized in medical education to assist diagnose and treating new patients. Figure 10 shows a general architecture of case-based reasoning. CBR research looks at the CBR process as a model of human cognition as well as a method for developing intelligent systems.

CBR is utilized in a variety of applications. Lamy et al. [52], for example, provide a visual case-based reasoning strategy for explainable artificial intelligence for breast cancer. Gonzalez et al. [30] provide a case-based reasoning-based energy optimization technique. Khosravani et al. [47] offers a case-based reasoning application in a defect detection system for dripper manufacturing. Corrales et al. [17] provide a case-based reasoning system for data cleaning algorithm recommendation in classification and regression problems. As the number of stored cases grows, CBR becomes more intelligent and thus might be useful in such scenarios while building a model. However, as the time required to find and process relevant cases increases, the system’s efficiency will decline.

### Text Mining and Natural Language Processing

Text mining [7], also known as text data mining, similar to text analytics, is the process of extracting meaningful information from a variety of text or written resources, such as websites, books, emails, reviews, docs, comments, articles, and so on. Information retrieval, lexical analysis to investigate word frequency distributions, pattern recognition, tagging or annotation, information extraction, and data mining techniques such as link and association analysis, visualization, and predictive analytics are all part of text analysis. Text mining achieves this by employing several analysis techniques, such as natural language processing (NLP). NLP is a text analysis technique that allows machines to interpret human speech. NLP tasks include speech recognition, also known as speech-to-text, word segmentation or tokenization, lemmatization and stemming, part of speech tagging, parsing, word sense disambiguation, named entity recognition, sentiment analysis, topic segmentation and recognition, and natural language generation, which is the task of converting structured data into human language [21]. Fake news identification, spam detection, machine translation, question answering, social media sentiment analysis, text summarization, virtual agents and chatbots, and other real-world applications use NLP techniques.

Although many language-processing systems were built in the early days using symbolic approaches, such as hand-coding a set of rules and looking them up in a dictionary, NLP now blends computational linguistics with statistical, machine learning, and deep learning models [80, 81]. These technologies, when used together, allow computers to process human language in the form of text or speech data and comprehend its full meaning, including the speaker’s or writer’s intent and sentiment. Many works have been done in this area. For example, using the feature ensemble model, Phan et al. [68] propose a method for improving the performance of sentiment analysis of tweets with a fuzzy sentiment. Using weighted word embeddings and deep neural networks, Onan et al. [62] provide sentiment analysis on product reviews. Subramaniyaswamy et al. [96] present sentiment analysis of tweets for estimating event criticality and security. In [60], the efficacy of social media data in healthcare communication is discussed. Typically, learning techniques rather than static analysis is more effective in terms of automation and intelligence in textual modeling or NLP systems. In addition to standard machine learning algorithms [81], deep learning models and techniques, particularly, based on convolutional neural networks (CNNs) and recurrent neural networks (RNNs) enable such systems to learn as they go and extract progressively accurate meaning from large amounts of unstructured, unlabeled text and speech input. Thus various deep learning techniques including generative and discriminative models can be used to build powerful textual or NLP model according to their learning capabilities from data, discussed briefly in our earlier paper Sarker et al. [80], which could also be a significant research direction in the area. Overall, we can conclude that by combining machine and deep learning techniques with natural language processing, computers can intelligently analyze, understand, and infer meaning from human speech or text, and thus could be useful for building textual AI models.

### Visual Analytics, Computer Vision and Pattern Recognition

Computer vision [99] is also a branch of AI that allows computers and systems to extract useful information from digital images, videos, and other visual inputs and act or make recommendations based on that data. From an engineering standpoint, it aims to comprehend and automate operations that the human visual system is capable of. As a result, this is concerned with the automated extraction, analysis, and comprehension of relevant information from a single image or a series of images. In terms of technology, it entails the creation of a theoretical and algorithmic foundation for achieving autonomous visual understanding by processing an image at the pixel level. Typical tasks in the field of visual analytics and computer vision include object recognition or classification, detection, tracking, picture restoration, feature matching, image segmentation, scene reconstruction, video motion analysis, and so on.

**Fig. 11:**
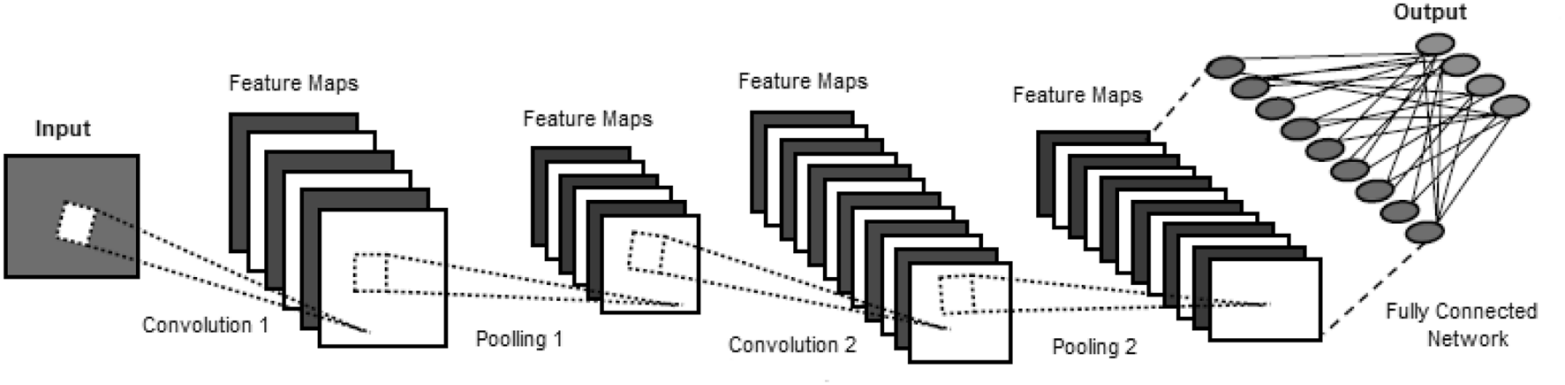
A general architecture of a convolutional neural network (CNN or ConvNet)

Pattern recognition, which is the automated recognition of patterns and regularities in data, is the basis for today’s computer vision algorithms. Pattern recognition often involves the categorization (supervised learning) and grouping (unsupervised learning) of patterns [81]. Although pattern recognition has its roots in statistics and engineering, due to the greater availability of huge data and a new wealth of processing power, some recent techniques to pattern recognition include the use of machines and deep learning. Convolutional neural networks (CNN or ConvNet) [53, 80] have recently demonstrated considerable promise in a variety of computer vision tasks, including classification, object detection, and scene analysis. The general architecture of a convolution neural network is depicted in Figure 11. Large datasets of thousands or millions of labeled training samples are typically used to train these algorithms. However, the lack of appropriate data limits the applications that can be developed. While enormous volumes of data can be obtained fast, supervised learning also necessitates data that has been labeled. Unfortunately, data labeling takes a long time and costs a lot of money. In this area, a lot of work has been done. Elakkiya et al. [25] develop a cervical cancer diagnostics healthcare system utilizing hybrid object detection adversarial networks in their paper. Harrou et al. [38] present an integrated vision-based technique for detecting human falls in a residential setting. Pan et al. [63] demonstrated a visual recognition based on deep learning for navigation mark classification. Typically, learning techniques rather than static analysis is more effective in terms of automation and intelligence in such visual analytics. In addition to standard machine learning algorithms [81], various deep learning techniques including generative and discriminative models can be used to build powerful visual model according to their learning capabilities from data, discussed briefly in our earlier paper Sarker et al. [80], which could also be a significant research direction in the area. Thus, this is important to build effective visual AI models in various application areas to solve real-world issues in the current age of the Fourth Industrial Revolution or Industry 4.0, according to the goal of this paper.

### Hybrid Approach, Searching, and Optimization

A “hybrid approach” is a blend of multiple approaches or systems to design a new and superior model. As a result, a hybrid strategy integrates the necessary approaches outlined above depending on the demands. For instance, in our earlier publication, Sarker et al. [85], we have used a hybridization of machine learning and knowledge-base expert system to build an effective context-aware model for intelligent mobile services. In this hybrid context-aware model, context-aware rules are discovered using machine learning techniques, which are used as the knowledge base of an expert system rather than traditional handcrafted static rules to make computing and decision-making processes more actionable and intelligent. Similarly, in another hybrid approach [68], the concepts of fuzzy logic, deep learning, and natural language processing were integrated to improve Twitter sentiment analysis accuracy. The authors in [33] present a deep convolutional neural network-based automated and robust object recognition in X-ray baggage inspection, where deep learning is integrated with computer vision analysis. Kang et al. [44] proposed a fuzzy weighted association rule mining strategy to produce a customer satisfaction product form. Moreover, Sarker et al. discussed various machine learning [81] and deep learning [80] techniques and their hybridization that can be used to solve a variety of real-world problems in many application areas such as business, finance, healthcare, smart cities, cybersecurity, etc. Thus, hybridization of multiple techniques could play a key role to build an effective AI model in the area.

Moreover, many AI problems can be solved theoretically by searching through a large number of possible solutions, and the reasoning process may be reduced down to a simple search. Thus, search strategies, also known as universal problem-solving approaches in AI, can also play a significant role to solve real-world issues such as gaming, ranking web pages, video, and other content in search results, etc., due to the properties of its completeness, optimality, time complexity, and space complexity. Depending on the nature of the problems, search algorithms can be uninformed search (a.k.a. blind, brute-force) or informed search (a.k.a. heuristic search). Uninformed search [75] refers to a group of general-purpose search algorithms that generate search trees without relying on domain information, such as breadth-first, depth-first, uniform cost search, etc. Informed search [75] algorithms, on the other hand, use additional or problem-specific knowledge in the search process, such as greedy search, A* search, graph search, etc. For example, when searching on Google Maps, one needs to provide information such as a position from the current location to precisely traverse the distance, time traveled, and real-time traffic updates on that specific route. Informed search can solve a variety of complicated problems that cannot be handled any other way. Furthermore, evolutionary computation employs an optimization search technique, such as genetic algorithms, which has a great potential to solve real-world issues. For instance, in the domain of cybersecurity, a genetic algorithm is used for effective feature selection to detect anomalies in fog computing environment [61]. In [28], genetic algorithm is used for optimized feature selection to detect Android malware using machine learning techniques. With AI-powered search, the platform learns from the data to provide the most accurate and relevant search results automatically. Thus, searching as well as optimization techniques can be used as a part of hybridization while building AI models to solve real-world problems.

Overall, we can conclude that the above explored ten potential AI techniques can play a significant role while building various AI models such as analytical, functional, interactive, textual, and visual models, depending on the nature of the problem and target application. In the next section, we summarize various real-world application areas, where these AI techniques are employed in today’s interconnected world towards automation, intelligent and smart systems.

**Fig. 12:**
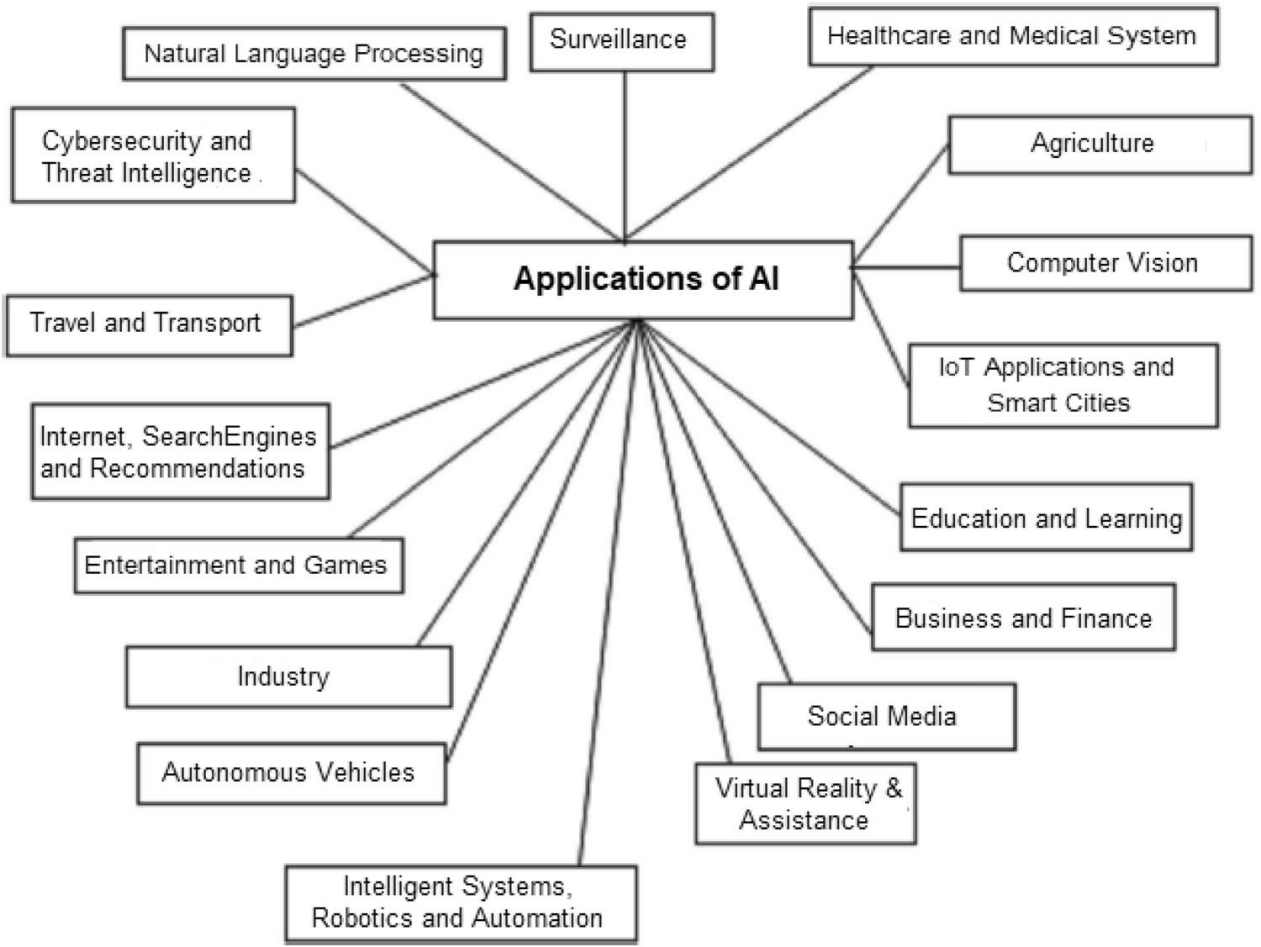
Several potential real-world application areas of artificial intelligence (AI)

**Table 3:** A summary of AI tasks and methods in several popular real-world applications areas

AI techniques	Application areas	Tasks	References
Machine learning	Healthcare Cybersecurity Smartcity Recommendation systems	COVID-19 aid Anomaly and Attack Detection Smart parking pricing system Hotel recommendation	Blumenstock et al. [12] Sarker et al. [78], Sarker et al. [89], Saharan et al. [76], Ramzan et al. [73]
Neural network and deep learnig	Healthcare Cybersecurity Smart cities Smart Agriculture Business and Finance Virtual Assistant Visual Recognition	Diagnosis of COVID-19 Malware detection Smart parking system Plant disease detection Stock trend prediction An intelligent chatbot Facial expression analysis	Aslan et al. [9], Islam et al. [41] Kim et al. [48], Wang et al. [100] Piccialli et al. [69] Ale et al. [6] Anuradha et al. [8] Dhyani et al. [22] Li et al. [54]
Data mining, knowledge discovery and advanced analytics	Education Business Cybersecurity Diagnostic analytics Prescriptive analytics	Decision support systems Maximising competitive advantage Human-centred data mining To mature gas fields Optimizing outpatient appointment	Hamed et al. [35] Alazab et al. [5] Afzaliseresht et al. [1] Poort et al. [70] Srinivas et al. [94]
Rule-based modeling and decision-making	Intelligent systems Healthcare Recommendation system Smart systems	Mining contextual rules Identifying risk factors Web page recommendation Risk prediction	Sarker et. al [88] Borah et al. [13] Bhavithra et al. [11] Xu et al. [103]
Fuzzy logic-based approach	Healthcare Agriculture Cybersecurity Business	Heart disease diagnosis Smart irrigation Network anomaly detection system Customer satisfaction	Reddy et al. [74] Krishnan et al. [51] Hamamoto et al. [34] Kang et al. [44]
Knowledge representation, Uncertainty reasoning and Expert system modeling	Smart systems cloud computing cybersecurity Mobile expert system	Smart traffic monitoring Ontology data access control Vulnerability management Personalized decision-making	Goel et al. [29] Kiran et al. [49] Syed et al. [97] Sarker et al. [90]
Case-based reasoning	Healthcare Smart cities Smart Industry Recommendation Systems	Breast cancer management Energy management Fault detection system Classification and regression tasks	Lamy et al. [52] Gonzalez et al. [30] Khosravani et al. [47] Corrales et al. [17]
Text mining and natural language processing	Sentiment analysis Business Cybersecurity Healthcare	Sentiment analysis of tweets Product reviews sentiment Estimating security of events Effectiveness of social media	Phan et al. [68] Onan et al. [62] Subramaniyaswamy et al. [96] Nawaz et al. [60]
Visual analytics, computer vision and pattern recognition	Healthcare Computer vision Visual Analytics	Cervical cancer diagnostics Human fall detection Navigation mark classification	Elakkiya et al. [25] Arrou et al. [38] Pan et al. [63]
Hybrid approach, searching and optimization	Mobile application Recommendation systems Sentiment analysis Business Cybersecurity	Personalized decision-making Personalized hotel recommendation Tweet sentiment accuracy analysis Customer satisfaction Optimum feature selection	Sarker et al. [90] Ramzan et al. [73] phan et al. [68] Kang et al. [44] Onah et al. [61], Fatima et al. [28]

## Real-World Applications of AI

AI approaches have been effectively applied to a variety of issues in a variety of application areas throughout the last several years. Healthcare, cybersecurity, business, social media, virtual reality and assistance, robotics, and many other application areas are common nowadays. We have outlined some potential real-world AI application areas in Fig. 12. Various AI techniques, such as machine learning, deep learning, knowledge discovery, reasoning, natural language processing, expert system modeling, and many others, as detailed above in “Potential AI techniques” are used in these application domains. We have also listed several AI tasks and techniques that are utilized to solve in several real-world application areas in Table 3. Overall, we can conclude from Fig. 12 and Table 3 that the future prospects of AI modeling in real-world application domains are vast and there are several opportunities to work and conduct research. In the following section, we discuss the future aspect of AI as well as research issues towards automation, intelligent and smart systems.

## Future Aspect and Research Issues

Artificial intelligence is influencing the future of almost every sector and every person on the planet. AI has acted as the driving force behind developing technologies for industrial automation, medical applications, agriculture, IoT applications, cybersecurity services, etc. summarized in “Future Aspect and Research Issues”, and it will continue to do so for the foreseeable future. This interdisciplinary science comes with numerous advancements and approaches that are possible with the help of deep learning, machine learning algorithms, knowledge-base expert systems, natural language processing, visual recognition, etc. discussed briefly in “Potential AI techniques”. Thus, by taking into account the capabilities of AI technologies, we illustrate three essential terms, mentioned in “Introduction” within the scope of our study. These are

- *Automation* One of the main themes of today’s applications is automation, which encompasses a wide range of technologies that reduce human interaction in operations. A program, a script, or batch processing are commonly used in computing to automate tasks. AI-based automation takes the insights gained through computational analytics to the next level, allowing for automated decision-making. As a result, we can describe automation as the development and implementation of technology to manufacture and deliver products and services to increase the efficiency, dependability, and/or speed of various jobs traditionally handled by humans. In customer service, for example, virtual assistants can lower expenses while empowering both customers and human agents, resulting in a better customer experience. Artificial intelligence technology has the potential to automate almost any industry and every person on the planet.
- *Intelligent computing* It is also known as computational intelligence, and it refers to a computer’s or system’s ability to extract insights or usable knowledge from data or experimental observation, or to learn a specific task. Intelligent computing methodologies include information processing, data mining, and knowledge discovery, as well as machine learning, pattern recognition, signal processing, natural language processing, fuzzy systems, knowledge representation, and reasoning. Transportation, industry, health, agriculture, business, finance, security, and other fields could all benefit from intelligent systems. Thus, the above-mentioned AI techniques, discussed in “Potential AI techniques” are the main drivers for performing intelligent computing as well as decision-making.
- *Smart computing* The word “Smart” can be described as self-monitoring, analyzing, and reporting technology in smart computing, and the word “Computing” can be defined as computational analysis. As a result, it can be thought of as the next generation of computing, which is used to create something self-aware, that is, something that can sense the activities of its environment, massage the gathered data, perform some analytics, and provide the best decisions while also predicting future risks and challenges. In other words, it is a significant multidisciplinary area in which AI-based computational methods and technologies, as explained in “Potential AI techniques”, are integrated with engineering approaches to produce systems, applications, and new services that suit societal demands. Overall, it strives to construct a smart system by monitoring, analyzing, and reporting data in a faster and smarter manner, with AI-based modeling playing a vital part in system intelligence and decision-making.

The above terms are also the key focus of the current fourth industrial revolution (Industry 4.0). Business, health care, energy, transportation systems, environment, security, surveillance, industrial systems, information retrieval and publication, entertainment and creativity, and social activities can all benefit from automation, intelligence, and smart computer systems. For example, chatbots, consumer personalization, image-based targeting advertising, and warehouse and inventory automation are all examples of how AI will continue to drive e-commerce. The potential benefits of using AI in medicine are now being investigated. The medical industry has a wealth of data that may be used to develop healthcare-related predictive models. Manufacturing, notably the automobile industry, will be significantly impacted by AI. AI will have an impact on sales operations in a range of industries. Marketing tactics, such as business models, sales procedures, and customer service options, as well as customer behavior, are predicted to be significantly influenced by AI. AI and machine learning will be key technologies in cybersecurity for identifying and forecasting threats [77, 89]. AI will be a vital tool for financial security because of its ability to analyze large amounts of data, foresee fraud, and identify it. In the near future, interacting with AI will surely become commonplace. Artificial intelligence can be used to solve incredibly difficult problems and find solutions that are vital to human well-being. These developments have enormous economic and societal implications. Thus, we can say, AI’s potential is limitless and its future will be shaped by our decisions and actions. While our discussion has established a solid foundation on AI-based systems and applications, hence we outline the below ten research issues.

- Several potential AI techniques exist in the area with the capability of solving problems, discussed in “Potential AI techniques”. To understand the nature of the problem and an in-depth analysis is important to find a suitable solution, i.e., detecting cyber-anomalies or attacks [78]. Thus, the challenge is “Which AI technique is most suited to solving a specific real-world problem, taking into account the problem’s nature?”
- One promising research direction for AI-based solutions is to develop a general framework that can handle the issues involved. A well-designed framework and experimental evaluation are both a crucial direction and a significant challenge. Thus, the question is “How can we design an effective AI-based framework to achieve the target outcome by taking into account the issues involved?”
- The digital world contains a wealth of data in this age of the Fourth Industrial Revolution (Industry 4.0 or 4IR), including IoT data, corporate data, health data, cellular data, urban data, cybersecurity data, and many more [79]. Extracting insights using various analytical methods is important for smart decision-making in a particular system. Thus, the question is “How to extract useful insights or knowledge from real-world raw data to build an automated and intelligent system for a particular business problem?
- Nowadays, data are considered as the most valuable resource in the world and various machine learning [81] and deep learning [80] techniques are used to learn from data or past experience, which automates analytical model building. The increase in data and such data-driven analytical modeling have made AI the highest growth in history. Thus, it’s important to do some data pre-processing tasks to feed into the ultimate machine learning model, so the data behaves nicely for the model. Therefore, the question is “How to effectively feed data to a machine or deep learning model to solve a particular real-world problem?”
- The traditional machine learning [81] and deep learning [80] techniques may not be directly applicable for the expected outcome in many cases. Thus, designing new techniques or their variants by taking into account model optimization, accuracy, and applicability, according to the nature of the data and target real-world application, could be a novel contribution in the area. Therefore the question is—“How to design an effective learning algorithm or model allowing the application to learn automatically from the patterns or features in the data?”
- In the domain of today’s smart computing, the term ‘context-awareness’ typically refers to a system’s capacity to gather information about its surroundings at any given time and adapt its behavior accordingly. Thus, the concept of context-aware machine learning can play a key role to build an intelligent context-aware application, highlighted in our book Sarker et al. [85]. Thus, the question is “How to effectively incorporate context-awareness in an AI-based smart system that can sense from the surrounding environment and make intelligent decisions accordingly?”
- Decision rules, represented as IF-THEN statement, can play an important role in the area of AI. Expert systems, a core part of AI, are typically used to solve many real-world complex problems by reasoning through knowledge, which are mostly represented by such IF-THEN rules rather than traditional procedural code [85]. Thus, a rule-based system can manipulate knowledge and interpret information in a useful way.  Therefore, the question is “How can we design an automated rule-based system emulating the decision-making ability of a human expert through discovering a concise set of IF-THEN rules from the data?
- A decision support system is a type of information system that aids in the decision-making process of a business or organization. AI techniques discussed in “Potential AI techniques” can play a key role to provide intelligent decisions across a wide range of sectors (e.g., business, education, healthcare, etc.) rather than the traditional system, according to the nature of the problem. Thus, the challenge is “How can we design an AI-assisted decision-support system that aids a team or organization in making better decisions?”
- Uncertainty refers to an event’s lack of confidence or certainty, e.g., information occurred from unreliable sources. Several strategies, such as the probability-based model or fuzzy logic, discussed in “Potential AI techniques” allow for the processing of uncertain and imprecise knowledge while also providing a sophisticated reasoning framework. The ability of AI to identify and handle uncertainty and risk is essential for applying AI to decision-making challenges. Thus, the question is “How to manage uncertainty in AI-enabled decision-making applications”.
- With the widespread availability of various IoT services, Internet of things (IoT) devices are becoming more common in mobile networks. It is essential nowadays to have a lightweight solution that promises high-performing artificial intelligence applications for mobile and IoT devices. Thus, the question is “How to design AI-enabled lightweight model for intelligent decision-making through IoT and mobile devices”.

To summarize, AI is a relatively open topic to which academics can contribute by inventing new methods or refining existing methods to address the issues raised above and solve real-world problems in a range of application areas. AI will be employed in any context where large amounts of data are needed to be handled fast and accurately, and cost savings are required. AI will affect the planet more than anything else in human history. One important thing is that AI-powered automation does not pose a threat to jobs in the workplace for individuals, businesses, or countries with the appropriate skills. AI-certified professionals have access to a wide range of job prospects. AI Engineer, Artificial Intelligence Programmer, AI System Developer, Data Scientist, Machine Learning Engineer, Data Analyst, AI Architect, Deep Learning Engineer, AI Software Engineer, and many other employment opportunities are available to these professionals.

Overall, AI technologies are driving a new wave of economic progress, resolving some of the world’s most challenging issues and delivering solutions to some of humanity’s most significant challenges. Many industries, including information technology, telecommunications, transportation, traffic management, health care, education, criminal justice, defense, banking, and agriculture, have the potential to be transformed by artificial intelligence. Without compromising the significant characteristics that identify mankind, we can assure that AI systems are deliberate, intelligent, and flexible with adequate security. Governments and decision-makers of a country need to focus public policies that promote AI innovation while minimizing unexpected societal consequences to realize its full potential in real-world scenarios.

## Concluding Remarks

In this article, we have provided a comprehensive view of AI-based modeling which is considered a key component of the fourth industrial revolution (Industry 4.0). It begins with research motivation and proceeds to AI techniques and breakthroughs in many application domains. Then in numerous dimensions, the important techniques in this area are explored. We take into account ten categories of popular AI techniques in this thorough analysis, including machine learning, deep learning, natural language processing, knowledge discovery, expert system modeling, etc., which can be applied in a variety of applications depending on current demands. In terms of machine intelligence, complex learning algorithms should be trained using data and knowledge from the target application before the system can help with intelligent decision-making.

Overall, AI techniques have proven to be beneficial in a variety of applications and research fields, including business intelligence, finance, healthcare, visual recognition, smart cities, IoT, cybersecurity, and many more, as explored in the paper. Finally, we explored the future aspects of AI towards automation, intelligence, and smart computing systems, highlighting several research issues within the scope of our study. This can also aid researchers in conducting more in-depth analyses, resulting in a more reliable and realistic outcome. Overall, we feel that our study and discussion on AI-based modeling points in the right direction and can be used as a reference guide for future research and development in relevant application domains by academics as well as industry professionals.
